# High Resolution Melt (HRM) analysis is an efficient tool to genotype EMS mutants in complex crop genomes

**DOI:** 10.1186/1746-4811-7-43

**Published:** 2011-12-08

**Authors:** Seosamh Ó Lochlainn, Stephen Amoah, Neil S Graham, Khalid Alamer, Juan J Rios, Smita Kurup, Andrew Stoute, John P Hammond, Lars Østergaard, Graham J King, Phillip J White, Martin R Broadley

**Affiliations:** 1 School of Biosciences, University of Nottingham, Sutton Bonington Campus, Loughborough LE12 5RD, UK; 2 Rothamsted Research, Harpenden AL5 2JQ, UK; 3 Department of Crop Genetics, John Innes Centre, Norwich, NR4 7UH, UK; 4 Southern Cross Plant Science, Southern Cross University, PO Box 157Lismore NSW 2480, Australia; 5 The James Hutton Institute, Invergowrie, Dundee, DD2 5DA, UK

**Keywords:** TILLING, HRM, Brassica, genotyping

## Abstract

**Background:**

Targeted Induced Loci Lesions IN Genomes (TILLING) is increasingly being used to generate and identify mutations in target genes of crop genomes. TILLING populations of several thousand lines have been generated in a number of crop species including *Brassica rapa*. Genetic analysis of mutants identified by TILLING requires an efficient, high-throughput and cost effective genotyping method to track the mutations through numerous generations. High resolution melt (HRM) analysis has been used in a number of systems to identify single nucleotide polymorphisms (SNPs) and insertion/deletions (IN/DELs) enabling the genotyping of different types of samples. HRM is ideally suited to high-throughput genotyping of multiple TILLING mutants in complex crop genomes. To date it has been used to identify mutants and genotype single mutations. The aim of this study was to determine if HRM can facilitate downstream analysis of multiple mutant lines identified by TILLING in order to characterise allelic series of EMS induced mutations in target genes across a number of generations in complex crop genomes.

**Results:**

We demonstrate that HRM can be used to genotype allelic series of mutations in two genes, *BraA.CAX1a *and *BraA*.*MET1.a *in *Brassica rapa*. We analysed 12 mutations in *BraA.CAX1.a *and five in *BraA*.*MET1.a *over two generations including a back-cross to the wild-type. Using a commercially available HRM kit and the Lightscanner™ system we were able to detect mutations in heterozygous and homozygous states for both genes.

**Conclusions:**

Using HRM genotyping on TILLING derived mutants, it is possible to generate an allelic series of mutations within multiple target genes rapidly. Lines suitable for phenotypic analysis can be isolated approximately 8-9 months (3 generations) from receiving M_3 _seed of *Brassica rapa *from the RevGenUK TILLING service.

## Background

The identification and analysis of gene mutations in plants is fundamental to the investigation of gene function. One approach, Targeted Induced Loci Lesions IN Genomes (TILLING) was originally developed in Arabidopsis [1] and has subsequently been successful in a range of crop plants [2,3]. In this reverse genetic approach, an ethyl methane sulfonate (EMS) mutagenised population is screened for SNPs within target genes [4]. EMS mutagenesis generates multiple alleles within each gene, including nonsense, missense, splicing and cis-regulatory mutants, in comparison to T-DNA and transposon mutagenesis that generate only knockout mutants [5,6]. Analysis of an allelic series can provide information on important domains or amino acids within the protein of interest. A number of TILLING populations have been developed for a variety of crops of different genome size, including rice [2], wheat [7,8], *Brassica rapa *[3], *Brassica napus *[9], *Lotus japonicus *[10], *Medicago trunculata *[11], *Arachis hypogaea *[12], and *Solanum lycopersicum *[13]. These populations have subsequently been used to isolate mutations in a variety of genes, including those involved in starch metabolism in *Lotus japonicas *[14], peanut allergens [12] and a gene encoding a fatty acid elongase [9].

High resolution melt (HRM) analysis is a technique that measures the disassociation of double-stranded DNA at high temperature resolution, and permits the analysis of genetic variations (SNPs, mutations, methylation) in PCR amplicons [15-17]. This technique allows genotyping and mutation scanning without the need for costly labeled probes, as it uses high fidelity heteroduplex-detecting double-stranded DNA binding dyes, such as EvaGreen which exhibit equal binding affinities for GC-rich and AT-rich regions and no sequence preference [18,19]. The heteroduplex products are detected by the presence of a second low-temperature melting transition [20]. This enables the reaction to be performed in a single tube, making it cost-effective and suitable for high-throughput screening [17]. HRM has been used extensively in the genotyping of human tissue samples for the identification of genes associated with diseases [21,22] as well as identification of clinically important fungal species [23]. HRM has been used for quantitative detection of adulteration with related species of pants used for medicinal purposes [24]. HRM has been used previously to identify mutations in TILLING populations of tomato [13], wheat [8] and Medaka [25]. In the tomato study, HRM was used to identify mutations using DNA pools from an EMS mutagenised population of plants. The wheat studies demonstrated that HRM can be used to isolate novel mutations in starch branching enzyme IIa (*SBEIIa*) genes from an EMS population [8] and in mutation scans in mixed PCR amplicons containing three homeologous gene fragments [19]. It was also demonstrated that homozygous and heterozygous individuals of a single mutation could be genotyped using HRM [19].

The genus *Brassica *includes the closest crop relatives of *Arabidopsis thaliana*, such as *B. rapa *(A-genome, 2n = 2x = 20, ~550 Mbp), which includes vegetable crops (e.g. turnip, Chinese cabbage) and oil-seed crops, *B. oleracea *(C-genome, 2n = 2x = 18, > 600 Mbp) which includes vegetable crops (cauliflower, broccoli, cabbage) and the amphidiploid *B. napus *(AC-genome, 2n = 4x = 38, ~1100 Mbp), which includes oil-seed crops (canola, oilseed rape) and swede. As with many crop plants *Brassica *genomes are complex arising from a series of duplication events that has resulted in most genes being present in multiple paralogous and homeologous copies [26,27]. A TILLING population has recently been generated in *B. rapa *to enable gene function to be analysed [3] and has previously been used to identify mutations in the *B.rapa *orthologue of the INDEHISCENT gene [28]. The aim of this study is to determine if HRM analysis can be used to genotype TILLING mutants in the crop species *B. rapa *and thereby to improve the efficiency of functional genomics approaches in this species.

## Results and Discussion

### Gene selection and TILLING

In order to demonstrate that HRM is suitable for efficient genotyping of mutant lines identified using TILLING, two target genes were chosen, *BraA.CAX1.a *and *BraA*.*MET1.a*. In *A. thaliana*, *CAX1 *has been shown to encode a calcium-proton antiporter [29] and *MET1 *has been shown to encode a DNA methyltransferase [30]. The *CAX1 *sequence from *A. thaliana *[Genbank: NM_129373.3] was used to identify the homologous sequence from *B. rapa *and from this sequence, 1.5 kb including the transcriptional start was used as the target for TILLING. To isolate *BraA*.*MET1.a *from *B. rapa *R-o-18, primers were designed based on the *B. rapa *Chiifu-401 sequence [Genbank:AB251937] [31]. These produced a 2110 bp DNA fragment covering 136 bp upstream of the translational start site together with the first intron and the first 407 bp of the second exon. Both sequences were submitted to the RevGenUK TILLING service, which resulted in 20 and 17 mutations being identified in *BraA.CAX1.a *and *BraA*.*MET1.a *respectively (Table 1). These mutations included silent, missense, nonsense and non-coding mutations.

**Table 1:** Nucleotide changes in *BraA.cax1*.*a *and *BraA.met1.a *TILLING mutants.

**Line**^**a**^	**Mutation Change**^**b**^	**Position (base-pair)**^**c**^	**Position (amino-acid)**^**d**^	**Type of mutation**^**e**^
*BraA.cax1.a-1*	G to A	881	-	Non-coding

*BraA.cax1.a-2*	C to T	860	-	Non-coding

*BraA.cax1.a-3*	G to A	545	2	Non-coding

*BraA.cax1.a-4*	G to A	771	77	Missense

*BraA.cax1.a-5*	C to T	494	-	Non-coding

*BraA.cax1.a-6*	G to A	517	-	Non-coding

*BraA.cax1.a-7*	G to A	673	44	Missense

*BraA.cax1.a-8*	G to A	599	19	Missense

*BraA.cax1.a-9*	G to A	645	35	Missense

*BraA.cax1.a-10*	C to T	736	65	Missense

*BraA.cax1.a-11*	G to A	795	85	Missense

*BraA.cax1.a-12*	C to T	708	56	Missense

*BraA.cax1.a-13*	C to T	689	49	Silent

*BraA.cax1.a-14*	C to T	661	40	Missense

*BraA.cax1.a-15*	C to T	658	39	Missense

*BraA.cax1.a-16*	C to T	421	-	Non-coding

*BraA.cax1.a-17*	G to A	926	-	Non-coding

*BraA.cax1.a-18*	C to T	818	92	Silent

*BraA.cax1.a-19*	C to T	732	64	Missense

*BraA.cax1.a-20*	C to T	690	50	Nonsense

*BraA.met1.a-1*	C to T	3516	248	Silent

*BraA.met1.a-2*	G to A	3334	188	Missense

*BraA.met1.a-3*	G to A	3334	188	Missense

*BraA.met1.a-4*	G to A	3084	104	Silent

*BraA.met1.a-5*	C to T	3081	103	Silent

*BraA.met1.a-6*	G to A	3252	243	Nonsense

*BraA.met1.a-7*	G to A	3500	243	Missense

*BraA.met1.a-8*	G to A	3012	80	Silent

*BraA.met1.a-9*	G to A	3495	241	Silent

*BraA.met1.a-10*	C to T	3335	188	Missense

*BraA.met1.a-11*	C to T	3069	99	Silent

*BraA.met1.a-12*	G to A	3330	186	Silent

*BraA.met1.a-13*	C to T	3324	184	Silent

*BraA.met1.a-14*	G to A	3213	147	Nonsense

*BraA.met1.a-15*	C to T	3265	165	Silent

*BraA.met1.a-16*	G to A	3330	186	Silent

*BraA.met1.a-17*	G to A	3258	162	Silent

^a ^- TILLING line, ^b ^- Nucleotide change in mutant line, ^c ^- Position of nucleotide change in base-pairs, ^d ^- Position of amino-acid change in peptide, ^e ^- Type of mutation caused by nucleotide change. Non-coding - nucleotide change not within coding region, missense - nucleotide change results in an amino-acid change, nonsense - nucleotide change results in the introduction of a premature stop codon, silent - no change in amino-acid.

### Genotyping of TILLING lines using HRM

**Figure 1:**
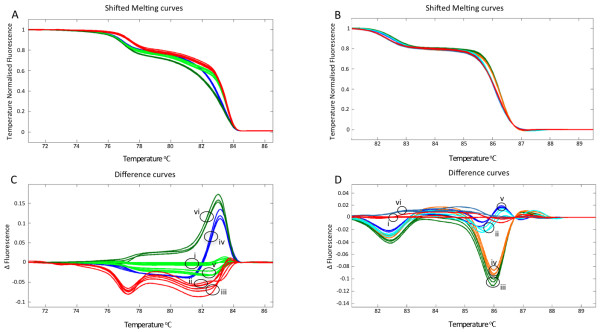
**HRM analysis for *Brassica rapa *R-o-18 wild-type and individual plants from M_3 _*BraA.cax1.a-11 *and *BraA.met1.a-6 *TILLING mutants**. A: Normalised melt curves for fluorescent signals from DNA strand dissociation of triplicated technical replicates of *B. rapa *wild-type (light green lines) and individual plants of M_3 _TILLING mutant *BraA.cax1.a-11*, plant *BraA.cax1.a-11A *(red), plant *BraA.cax1.a-11B *(red), plant *BraA.cax1.a-11C *(blue), plant *BraA.cax1.a-11D *(light green), plant *BraA.cax1.a-11E *(dark green). B: Normalised melt curves for fluorescent signals from DNA strand dissociation of triplicated technical replicates of *B. rapa *wild type (red lines) and individual plants M_3 _TILLING mutant *BraA.met1.a-6*, plant *BraA.met1.a-6-1 *(light blue), plant *BraA.met1.a-6-2 *(green), plant *BraA.met1.a-6-3 *(orange), plant *BraA.met1.a-6-4 *(purple), plant *BraA.met1.a-14-1 *(blue). C: Difference plot of genotypes' fluorescence normalised to wild type samples (light green lines, i) and individual plants of M_3 _TILLING mutant *BraA.cax1.a-11*, plant *BraA.cax1.a-11A *(red, ii), plant *BraA.cax1.a-11B *(red, iii), plant *BraA.cax1.a-11C *(blue, iv), plant *BraA.cax1.a-11D *(light green, v), plant *BraA.cax1.a-11E *(dark green, vi). D: Difference plot of genotypes' fluorescence normalised to wild type (red, i) and individual plants of the M_3 _TILLING mutant *BraA.met1.a-2*, plant *BraA.met1.a-6-1 *(light blue, ii), plant *BraA.met1.a-6-2 *(green, iii), plant *BraA.met1.a-6-3 *(orange, iv), plant *BraA.met1.a-6-4 *(purple, v), plant *BraA.met1.a-14-1 *(blue, vi).

**Figure 2:**
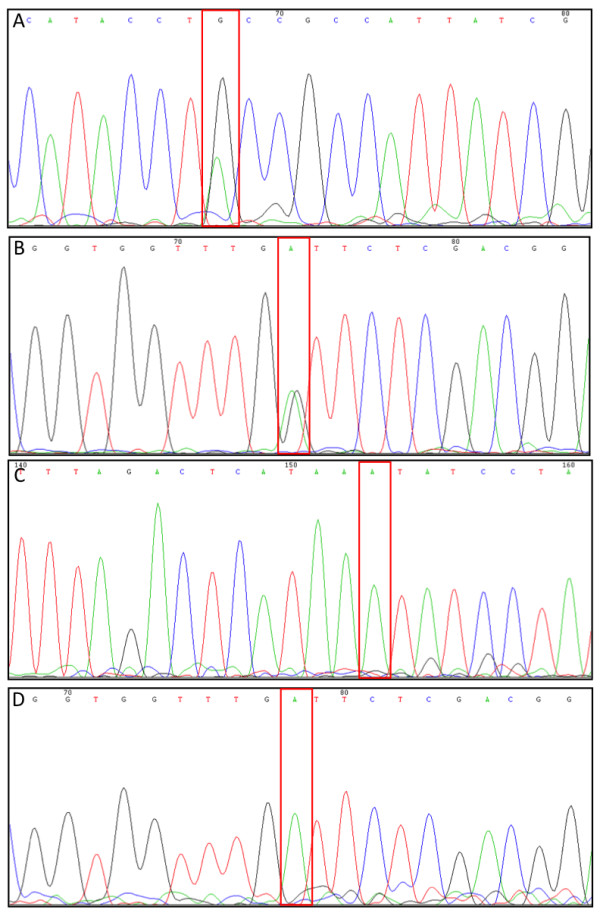
**Sequencing chromatograms of M_3 _generation of *B. rapa *R-o-18 mutants**. A: *BraA.cax1.a-11*, gene sequence from 788 bp to 807 bp shown. B: *BraA.met1.a-6*, gene sequence from 3246 bp to 3265 bp shown. C: *BraA.cax1.a-1*, gene sequence from 868 bp to 888 bp shown. D: *BraA.met1.a-6*, gene sequence from 3246 bp to 3265 bp shown. Rectangular box indicates zygosity state at the site of EMS induced transitional mutation.

From the lines identified using TILLING, twelve (*BraA.cax1.a-1, -4, -6*, -*7, -8, -10, -11, -12, -14, -16, -18, -20*) and five (*BraA*.*met1.a-3, -6, -7, -10 *and *-14*) lines carrying mutations in *BraA.CAX1 *and *BraA*.*MET1.a *respectively were chosen to be characterised based on the nature and position of the mutation. In order to genotype the lines, a HRM assay was developed for each gene. To increase the specificity of the HRM reaction a nested PCR approach was used for the *BraA.cax1.a *lines, first using the TILLING primers to amplify genomic DNA, prior to using the HRM primers with this product. In total, three primer pairs were designed to genotype all 12 mutations within the *BraA.CAX1.a *gene (amplifying fragments of 200, 203 and 260 bp; Table 2) and one primer pair was designed to genotype all the mutations within the *BraA*.*MET1.a *gene (amplifying a 399 bp fragment; Table 2). Individual M_3 _plants of the TILLING lines and wild-type R-o-18 plants were then genotyped using the HRM assay using a single primer pair per plate and three technical replicates per plant, allowing multiple alleles to be genotyped in a single scanning run. Analysis of the difference plots for the fluorescent signals detected individual plants that were wild-type, homozygous or heterozygous for the mutation in alleles of both genes (Figure 1). Three distinct groups can readily be observed in the difference plots. To confirm these results and assign zygosity groups, the PCR products from selected lines were sequenced. Sequencing the HRM PCR products identified by HRM as heterozygous from *BraA.cax1.a-11 *and *BraA.met1.a-6*, showed a double peak at the position of the mutation (Figure 2), confirming that these were indeed heterozygous for the mutations. In addition, sequencing of PCR products from *BraA.cax1.a-1 *and *BraA.met1.a-6 *identified as homozygous by HRM, confirmed that they were homozygous for the mutation (Figure 2). These results demonstrate that HRM can be used both for rapid genotyping of TILLING mutants and for distinguishing wild-type, heterozygous and homozygous plants. The results also demonstrate that multiple alleles can be genotyped using a single primer pair on one PCR plate, allowing multiple lines and individual plants to be genotyped simultaneously.

**Table 2:** Details of primers used for gene isolation and HRM analysis.

**Primer**^**a**^	**Gene**^**b**^	**Stage**^**c**^	**Mutation Targeted**^**d**^	**Sequence 5' to 3'**^**e**^
BrCAX1 TILLING_Forward	*BraA.CAX1.a*	TILLING		AGAGATTTCCTAGCCATGTG

BrCAX1 TILLING_Reverse	*BraA.CAX1.a*	TILLING		CGACCCCTAATTGTTTTATGTG

BrCAX1 HRM1_Forward	*BraA.CAX1.a*	HRM	6,16	TCCTCGAAGTTGCCTCTGAT

BrCAX1 HRM1_Reverse	*BraA.CAX1.a*	HRM	6,16	GCTGCTGACCATTGTTCCTG

BrCAX1 HRM3_Forward	*BraA.CAX1.a*	HRM	7,8,12,14,20	GCAGGAACAATGGTCAGCAG

BrCAX1 HRM3_Reverse	*BraA.CAX1.a*	HRM	7,8,12,14,20	CGAGAATGACTTCTTGGAGATT

BrCAX1 HRM6_Forward	*BraA.CAX1.a*	HRM	4,10,11,18	TCCAGAAGGTTCCATACAAAGG

BrCAX1 HRM6_Reverse	*BraA.CAX1.a*	HRM	4,10,11,18	CGCTGTTCTGTTTAGTAATGTGTTG

BrMet1aF4	*BraA.MET1.a*	Gene isolation		ACTTCACAGCATCTCCTCAGGGC

BrMet1aF5	*BraA.MET1.a*	Gene isolation		CTTGACAATGGTGCTGTTATTCAG

BrMet1aF6	*BraA.MET1.a*	Gene isolation		GTCTGGTTTGATGGCAGAGGACG

BrMet1aR4	*BraA.MET1.a*	Gene isolation		CGCACTTCGCAGCTTCAGAAAC

BrMet1aR5	*BraA.MET1.a*	Gene isolation		AAATAGAGATTGTTGAACACCCCC

BrMet1aR6	*BraA.MET1.a*	Gene isolation		TTGGATACCACAGGTGCCATGC

BrMet1aHRMF	*BraA.MET1.a*	HRM	3,6,7,10,14	TGCGATGATAAGGAGAAAGG

BrMet1aHRMR	*BraA.MET1.a*	HRM	3,6,7,10,14	TTTGCCGTCTCATCCAAAC

**^a^**- Primer name, ^b^-gene primer designed to, ^c^-stage of analysis primer used, ^d^-line primers used to genotype, ^e^- sequence of primer

### Backcross analysis

To reduce the mutation load, reciprocal crosses of the TILLING lines and wild-type R-o-18 plants were performed with both homozygous and heterozygous mutant plants. A heterozygous plant from the *BraA.met1.a-6 *line was crossed to a wild-type plant and ten F_1 _plants were genotyped. The results from the genotyping showed the expected segregation of the mutant allele, with 5 plants having a heterozygous mutation and 5 plants being wild-type for the mutation. The genotyping results for the F_1 _plants from crosses of the *BraA.cax1.a *lines also showed the expected genotyping results (Table 3). For example, the F_1 _plants of a cross using a homozygous *BraA.cax1.a-1 *plant were all heterozygous for the mutation (n = 5). In contrast, the F_1 _progeny of heterozygous plants showed segregation for the mutation (Table 3). These results demonstrate that HRM can be used to follow mutations and zygosity through multiple generations and crossing events, making it an ideal method for genotyping of TILLING mutants.

**Table 3:** HRM genotyping results of F_1 _progeny of *BraA.cax1*.*a *and *BraA.met1.a *lines crossed to wild-type.

**Line**^**a**^	**Plant**^**b**^	**Number of heterozygous plants**^**c**^	**Number of wild-type plants**^**d**^
*BraA.cax1.a-1*	E	5	0

*BraA.cax1.a-4*	C	2	0

*BraA.cax1.a-6*	B	4	2

*BraA.cax1.a-7*	A	3	3

*BraA.cax1.a-8*	A	4	1

*BraA.cax1.a-10*	A	3	2

*BraA.cax1.a-11*	B	3	3

*BraA.cax1.a-12*	A	3	3

*BraA.cax1.a-14*	B	3	0

*BraA.cax1.a-18*	D	2	4

*BraA.cax1.a-20*	A	2	2

*BraA.met1.a-6*	A	5	5

^a ^- TILLING line crossed to *B. rapa *R-o-18 wild-type, ^b ^- Individual M_3 _plant identifier' ^c ^- Number of individual F_1 _heterozygous plants identified by HRM' ^d ^- Number of individual F_1 _wild-type plants identified by HRM

## Conclusions

To analyse an allelic series of mutants generated using the TILLING approach efficiently, a cost effective, high-throughput genotyping technique is required. Techniques that have been used previously include PCR-RFLP, but this relies on the mutation causing a change in a restriction site [32] and TaqMan assays, which can require expensive probes and assay optimisation [18,33]. Alternatively, PCR products covering the mutations could be sequenced. However, this is time consuming and potentially more expensive; HRM analysis of samples in triplicate costs ~30% of Sanger sequencing costs. The results presented here demonstrate that HRM can be used to genotype mutations identified by TILLING from EMS populations in *B. rapa*. The results also demonstrate that a single PCR primer pair could be used to identify mutations at multiple positions with the PCR fragment. This reduces the number of primer pairs needed to genotype an allelic series and hence the cost. Due to the nature of the HRM process, the DNA extraction, PCRs and melt curve analysis can all be performed in 96-well plates. This enables high-throughput genotyping of multiple alleles of a number of genes to be performed quickly and efficiently. This is particularly important in complex crop species such as *B rapa*, where paralogous genes may be present and accumulation of mutations may be required to generate a phenotype.

## Methods

### Plant material

Seed of the highly inbred, homozygous, self-compatible, rapid cycling *B*. *rapa *ssp. *trilocularis *line R-o-18 was used as wild-type control. Seeds for lines (M_3 _generation) containing mutations in target genes were obtained from the R-o-18 TILLING population through the RevGenUK service [3,34].

### Plant growth conditions

Plants were grown under glasshouse conditions with a 16 h photoperiod at 22.3 ± 4°C and 13.3 ± 2°C mean day and night temperatures respectively. Seeds were sown directly into pots (height 11.3 cm; diameter 13 cm) containing Levington M3 high nutrient compost (Monro Group, Chichester, UK) and irrigated twice daily. Following three weeks of vegetative growth, Vitafeed^® ^2-1-4 nutrient solutions (N-P-K: 16-8-32 + micronutrients; Vitax Ltd., Coalville, Leicestershire, UK) were applied to plants weekly at a rate of 3 g l^-1 ^via overhead irrigation. Plants initiated flowering after approximately 6 weeks of growth and seeds were collected approximately 4 weeks thereafter. Perforated bread bags (150 mm × 700 mm, WR Wright & Sons Ltd, Liverpool, UK) were used to enclose inflorescences to prevent cross-pollination from neighboring plants.

### *In silico *data mining to identify gene sequences

Basic Local Alignment Search Tool (BLAST) [35] software was employed to identify sequences from all *Brassica *spp. that were orthologous to the *A*. *thaliana CAX*1 gene [Genbank: NM_129373.3]. All *A. thaliana *sequences of interest were retrieved from GenBank [36]. For the BLASTn algorithm [37] the entire GenBank database (March 2009) was interrogated to identify all *Brassica *sequences sharing > 80% sequence identity at an e-value of < 1e^-30 ^with the *A. thaliana *query sequence. The *Brassica *Genome Sequencing Project (*Br*GSP) database [38] was interrogated (March 2009) using the WuBLAST [39] algorithm for all *Brassica *BAC-end sequences, fully sequenced BACs, ESTs and genome survey sequence which shared > 80% sequence identity with this query sequence. *A. thaliana *genes sharing high sequence similarities to the query gene *i.e*. members of the same gene family including *AtCAX2 *[Genbank:NM_001036119.1] and *AtCAX3 *[Genbank:NM_115045.3] were used to interrogate both databases. After *in silico *comparative sequence analyses, *Brassica *sequences were assigned to the member of the *A. thaliana *gene family with which they shared the highest sequence identity, thus removing false positives. All selected sequences which were orthologous to *AtCAX1 *were formatted into FASTA and entered into a contig assembly program (ContigExpress, VectorNTI 11, Invitrogen, Paisley, UK) using default settings to form longer contiguous *Brassica *sequences and identify possible locus specific paralogues. These nascent *Brassica *gene sequences were aligned to the *A*. *thaliana *gene of interest, flanked at either end with 1 Mbp of sequence, using AlignX software at default settings. The genomic structure of these contigs was elucidated and annotated in the Vector map software (VectorNTI 11). Primers (Table 2) were designed to amplify ~ 1.2 kbp fragments of *BraA.CAX1.a *beginning upstream from the deduced transcriptional start site to a region within the gene using Primer 3 (Version 0.4.0) [40] with the parameters "Max Self Complementarity" and "Max 3' Self Complementarity" adjusted to 2, to avoid hairpin loops and potential dimerisation. Primers to amplify the *BraA*.*MET1.a *gene (Table 2) from *B. rapa *R-o-18 were designed based on the sequence described by Fujimoto et al. [[31], Genbank:AB251937].

### Amplification of locus specific sequences from *B. rapa *R-o-18

DNA was extracted from leaves of wild-type *B. rapa *R-o-18 using the GenElute^® ^Plant Genomic DNA Miniprep kit (Sigma-Aldrich, Gillingham, UK) according to the manufacturer's instructions and subjected to PCR amplification using primers (Table 2) designed to *Brassica *contig sequence. Amplified fragments were ligated in plasmid pCR8^®^/GW/TOPO^® ^(Invitrogen, Paisley, UK) and subsequently sequenced.

### Plant genomic DNA extraction

Genomic DNA was extracted from the *BraA*.*met1.a *plants using the DNeasy Plant Min kit (Qiagen, Crawley, UK) following the manufacturer's instructions or using a rapid salt-extraction method [41]. For the *BraA.cax1.a *plants, a high throughput extraction method was developed based on a method developed by Dr. M. Poole (pers. comm.). A 0.25 cm^2 ^diameter leaf disc from a developing or mid-expansion leaf from each plant was placed in a 96-well plate (Qiagen Collection Microtubes, Qiagen, Crawley, UK), with a single 3 mm stainless steel ball and a 600 μl aliquot of DNA extraction buffer (200 mM Tris-HCL pH 7.5, 250 mM NaCl, 25 mM EDTA and 0.5% w/v SDS) added to each well. Suspensions were shaken for 3 min in a ball mill (8000 Mixer Mill, Glen Creston, Stanmore, UK) until the majority of tissue was disrupted. Plates were subsequently centrifuged in a plate centrifuge (Centrifuge 5804, Eppendorf UK, Cambridge, UK) at 5,600 g for 20 min. After centrifugation 300 μl of the supernatant was transferred to a fresh plate (Thermofisher storage plate with rubber sealing mat, Fisher, Loughborough, UK), containing 300 μl of 100% isopropanol, mixed and incubated for 3 min at room temperature. Plates were then centrifuged at 5600 g for 20 min before discarding the supernatant. A 100 μl aliquot of 70% (v/v) ethanol was added and mixed by shaking vigorously for 5 min, centrifuged at 5,600 g for 20 min. The supernatant was discarded and dried in a vacuum manifold for 10 min. DNA was resuspended in 20 μl sterile H_2_O. The DNA concentration was not quantified and standardized due to the number of samples extracted simultaneously.

### High Resolution Melt (HRM) analysis

Primers were designed for HRM analysis to amplify 100-250 bp regions within the *BraA.CAX1*.*a *gene and a 399 bp region of *BraA*.*MET1.a*, which were originally used to identify mutations by TILLING. Three sets of HRM primer pairs were designed for *BraA.CAX1*.*a *and one for *BraA*.*MET1.a *(Table 2), using Primer 3 (Version 0.4.0) with the parameters "Max Self Complementarity" and "Max 3' Self Complementarity" adjusted to 2, and a T_m _≥ 60°C, to avoid hairpin loops and dimerisations. Primer sequences were examined further by primer thermodynamic software (Vector NTI 11) to confirm that no secondary structures were likely to form. HRM primer amplification efficiencies and specificities, were determined *in vitro *by PCR amplification of wild-type *B. rapa *R-o-18 DNA in reaction volumes containing 10 μl of GoTaq buffer (5×), 5 μl of MgCl_2 _(25 mM), 1 μl of dNTPs (10 mM), 5 μl of both forward and reverse primers (10 pmol μl^-1^), DNA (10 ng), 0.2 μl of Taq polymerase (GoTaq) and sterile de-ionised water (SDW) to a total volume of 50 μl. PCR conditions were 95°C for 5 min and 30 cycles of 95°C for 10 sec, 55°C for 10 sec and 72°C for 30 sec.

### Nested PCR approaches to amplify locus specific fragments for HRM analysis

The nested PCR approach was used for the *BraA.CAX1.a *lines. DNA extracted from TILLING lines were amplified with TILLING primers using Phusion™ high fidelity DNA polymerase (New England Biolabs, Hitchin, UK) to ensure sequence integrity of amplified sequences. Reaction volumes comprised of 4 μl of Phusion HF (5×), 0.4 μl of dNTPs (10 mM), 1.4 μl of forward and reverse *BraA.CAX1*.*a *TILLING primers (10 pmol μl^-1^), 1 μl DNA, 0.6 μl of DMSO, 0.2 μl of Phusion DNA polymerase and SDW to a total volume of 20 μl. PCR conditions involved an initial denaturation at 98°C (30 sec) followed by 30 cycles of denaturation at 98°C (5-10 sec), annealing at 58°C (15 sec) and extension at 72°C (30 sec).

### HRM reactions

HRM were performed using Type-it^® ^HRM™ PCR kit (Qiagen, Crawley, UK) following manufacturer's instructions; 1 μl of the initial nested PCR reaction for the *BraA.cax1.a *lines and 1 μl of genomic DNA for the *BraA.met1.a *lines was added as template to the HRM PCR reaction mix comprising 5 μl of 2 × HRM PCR Master Mix (including EvaGreen fluorescent dye), 1.75 μl of forward and reverse HRM primers (10 pmol μl^-1^) and SDW to 10 μl. Using an Eppendorf 96-well MasterCycler 5331, reaction volumes were subjected to 95°C (5 min), followed by 30 cycles of 95°C (10 sec), 55°C annealing (10 sec) and 72°C extension (30 sec). Included within each 96-well analysis plate were samples in triplicate, also triplicate wild-type DNA samples and two sets of negative controls, one without DNA added and one without HRM primer added.

### Lightscanner™ analysis of HRM products

PCR plates containing HRM amplicons were placed into a Lightscanner™ (Idaho Technology, Salt Lake City, USA) and the temperature raised to 60°C for 5 mins to ensure all samples were equilibrated. The melt temperature range was set at 63.3°C-95.4°C with a ramp setting at 0.1°C and a second hold at each step. Exposure was set at "Auto", background correction to exponential, curve shift to 0.020, standards to 'Auto Group', and sensitivity at normal +2.8. HRM analysis was then performed on the dissociation of double-stranded DNA PCR products, which had been saturated with the low PCR-toxic dye, EvaGreen from the initial HRM PCR. The LightScanner™ Data Analysis software (Version 2.0, Idaho Technology) was used to analyse the data and produce normalised disassociation curves and difference plots.

## Competing interests

The authors declare that they have no competing interests.

## Authors' contributions

SÓL developed and performed the *BraA-cax1.a *analysis and took the co-lead role in writing the manuscript. SA developed and performed the *BraA-met1.a *analysis. NG performed the *BraA-cax1.a *analysis and took the co-lead role in writing the manuscript. KA and JJR performed the *BraA-cax1.a *analysis. AS and SK developed the HRM analysis. JPH, LØ, GJK, PJW and MRB conceived and participated in development and coordination of the project. All authors contributed to the editing of the manuscript. All authors have read and approved the final manuscript.
